# Novel Insights into the Regulation of GnRH Secretion in Sheep Hypothalamic Nerve Cells by the *GRM1* Gene

**DOI:** 10.3390/ijms27094046

**Published:** 2026-04-30

**Authors:** Manjun Zhai, Wenlong Zheng, Zongsheng Zhao, Yifan Xie

**Affiliations:** 1 College of Animal Science, Xinjiang Agricultural University, Urumqi 830052, China; 13699980930@163.com; 2 College of Animal Science and Technology, Shihezi University, Shihezi 832003, China; zhaozongsh@shzu.edu.cn (Z.Z.); 18899593750@163.com (Y.X.)

**Keywords:** Kazakh sheep, *GRM1*, hypothalamic nerve cells, GnRH

## Abstract

Seasonal estrus limits sheep farming efficiency, making enhanced reproductive capacity and year-round estrus crucial for efficient breeding. *GRM1* could modulates neuronal signals and stimulates neuron excitability. Our preliminary transcriptomic analysis of hypothalamic tissues from Kazakh ewes during nonbreeding season anestrus and nonbreeding season nutritionally induced estrus strongly suggested that *GRM1* is a candidate gene that regulates estrus. The role of *GRM1* in sheep estrus remains to be investigated. *GRM1* expression was measured in hypothalamic tissues of Kazakh sheep during nonbreeding anestrus and nutritionally induced estrus via qPCR and immunohistochemistry. *GRM1*’s regulatory role in GnRH secretion and gene expression was studied in hypothalamic neurons via overexpression and RNAi. GnRH secretion changes were quantified by ELISA. *GRM1* mRNA expression was significantly increased in the hypothalamus of estrous Kazakh sheep, as confirmed by immunohistochemical staining. The results of hypothalamic neuron experiments revealed that the expression of *GRM1* was significantly upregulated after overexpression, which affected the expression of GnAQ, ITPR1, PLCB1 and PRKCB and ultimately promoted the secretion of GnRH. The expression of GnRH decreased after the interference in *GRM1* expression. *GRM1* modulates the secretion of GnRH in the hypothalamic nerve cells of Kazakh sheep through the glutamatergic synapse–calcium signaling pathway.

## 1. Introduction

Sheep farming stands as a vital cornerstone of animal husbandry in China. Sheep breeds display remarkable diversity, encompassing breeds exhibiting year-round estrus, such as Hu sheep and small-tailed Han sheep, alongside distinct breeds that exhibit seasonal estrus, such as Kazakh sheep, Altai sheep, and China Merino sheep. The Kazakh sheep ranks among the three major coarse wool sheep breeds in China and stands as a highly representative, locally excellent breed in Xinjiang. Favored by herders, it thrives on coarse feed, exhibits robust resilience against harsh conditions, and yields exceptionally delicious meat with a distinct flavor. However, its pronounced seasonal estrus severely constrains reproductive efficiency. Seasonal estrus in sheep is precisely orchestrated by the hypothalamic–pituitary–ovarian (HPO) axis, which is intricately synchronized with shifting environmental light [1]. Seasonal estrus is a comprehensive manifestation of the adaptability of ewes to various external phenomena. Under identical latitudinal conditions, variations in feeding management and nutritional status can cause different sheep breeds or different individuals of the same breed to exhibit estrus during the nonbreeding season [2]. The primary determinant underlying alterations in the estrus cycle is genetic factors. Seasonal estrus remains a major constraint hindering the advancement of the sheep breeding industry. Enhancing fertility is pivotal for developing highly efficient sheep breeding operations. Consequently, achieving year-round estrus in sheep has significant practical implications for production.

Metabotropic glutamate receptor 1 (*GRM1*) is a member of the metabotropic glutamate receptor (mGluR) family, which contains 8 identified subtypes (*GRM1*–GRM8). The *GRM1* gene (ENSOARG00020084779) is located on sheep chromosome 8 (OAR 8), spanning from position 70,490,918 to 70,925,319. It consists of 12 exons and 11 introns and is expressed primarily in brain tissue [3]. As a key component of the metabotropic glutamate receptor family, *GRM1* facilitates glutamate-induced excitatory neurotransmission through activation of its intrinsic G protein-coupled mechanism. This activation engages downstream G protein effectors and subsequent intracellular second messenger cascades, which collectively regulate neuronal excitability via a series of signaling events [4,5]. Current research on *GRM1* is primarily directed toward delineating its involvement in the pathogenesis of psychiatric and neurodevelopmental disorders [6,7]. Reports in the literature indicate that glutamic acid can promote the secretion of GnRH [8]. Following the co-culture of GnRH nerve cells with astrocytes, the expression of *GRM1* on GnRH nerve cells is significantly upregulated, and the secretion of GnRH is significantly increased [9]. Transcriptomic analysis of laying hens with different energy intakes during the growth period revealed that the *GRM1* gene is involved in the development of reproductive organs and estrogen production in poultry through the estrogen signaling pathway [10]. Comparative transcriptomic analyses of the gonadal axis in Bamei sheep with varying litter sizes revealed that the *GRM1* gene and the neural activity-related ligand–receptor interaction signaling pathway were involved, indicating that this gene and its associated signaling pathway may play a significant role in the regulation of oocyte number and follicular development through the gonadal axis [11].

Currently, there have been no reports on the role of the *GRM1* gene in sheep estrus. Based on transcriptomic findings from our group [12], we previously reported a potential role for *GRM1* in the molecular regulation of sheep estrus. Therefore, using quantitative real-time PCR and immunohistochemistry, we determined the spatial and temporal expression patterns of *GRM1* mRNA and protein in the hypothalamic tissues of Kazakh sheep in estrus and nutrient-induced estrus. Through gene overexpression and RNA interference (RNAi), the regulatory effect of *GRM1* on the secretion of GnRH in hypothalamic nerve cells at the cellular level, as well as its effects on the expression levels of estrus-related genes in sheep, were elucidated. This study aimed to investigate the role of *GRM1* in the regulation of GnRH secretion in Kazakh sheep and differences in its expression in hypothalamic tissues during the estrus and anestrus periods. This study reveals the molecular regulatory mechanisms of *GRM1* in estrus in sheep.

## 2. Results

### 2.1. Expression of GRM1 in Hypothalamic, Pituitary, and Ovarian Tissue in Kazakh Sheep

**Figure 1:**
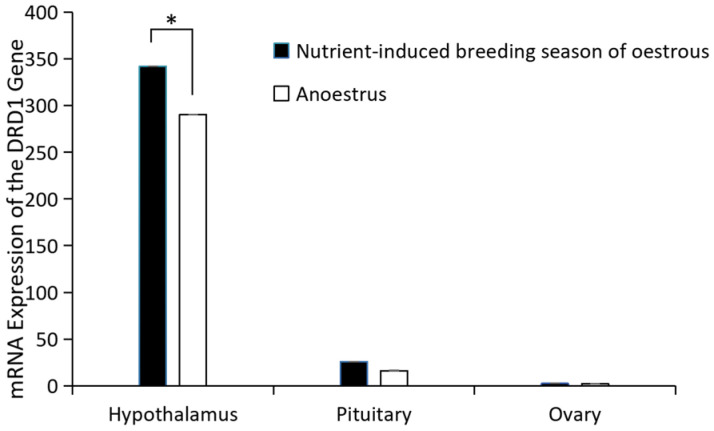
mRNA expression of *GRM1*. * *p* < 0.05.

*GRM1* gene expression in the hypothalamus, pituitary gland, and ovary of Kazakh sheep was detected by real-time fluorescent qPCR. The expression of *GRM1* mRNA in hypothalamic tissue was significantly higher than in pituitary and ovarian tissues, and exhibited higher expression during the estrous period compared to the non-estrous period, with these differences being highly significant (*p* > 0.05) (Figure 1).

### 2.2. Distribution of GRM1 Expression in Hypothalamic, Pituitary, and Ovarian Tissues

**Figure 2:**
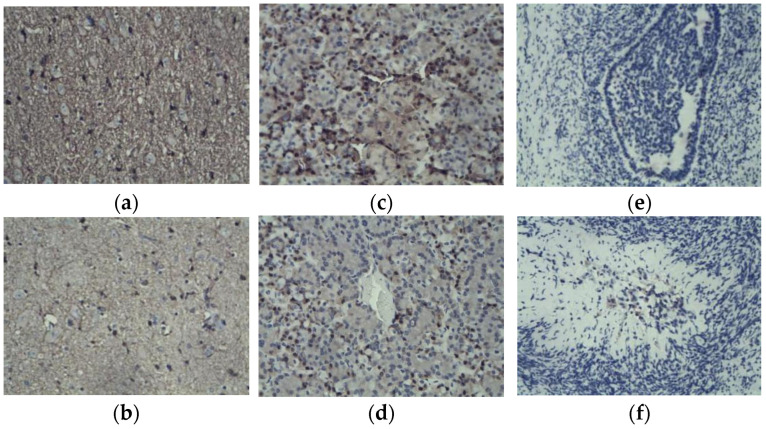
Immunohistochemical staining of hypothalamic (**a**,**b**), pituitary (**c**,**d**), and ovarian (**e**,**f**) tissue.

Immunohistochemical localization of *GRM1* expression in the hypothalamic, pituitary, and ovarian tissues of Kazakh sheep (Figure 2): *GRM1* was highly expressed in hypothalamic tissue, with positive cells (brown) forming a distinct dense network after staining. Staining was strongly positive during estrus (Figure 2a) and weakly positive during anestrus (Figure 2b). Fewer positive cells were detected in the pituitary tissue, and there were irregular round clusters and positive cytoplasmic staining. Compared with the estrus group (Figure 2c), the anestrus group showed fewer positive cells and tight cell clusters (Figure 2d). Histological examination of ovarian tissues revealed a near absence of positively stained cells during estrus (Figure 2e), whereas sporadic positive staining was detectable during anestrus (Figure 2f).

### 2.3. Assessment of GRM1 siRNA Efficacy

**Figure 3:**
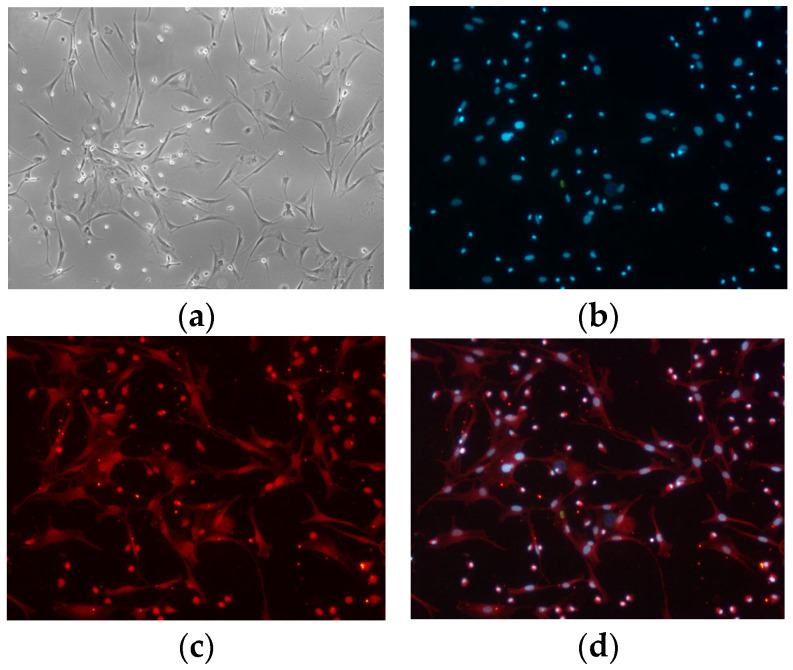
Culture, and identification of primary hypothalamic nerve cells. (**a**) Hypothalamic nerve cells. (**b**–**d**) Anti-MAP2-CY3 Immunofluorescence.

The isolation and culture of hypothalamic nerve cells were conducted in accordance with methodologies previously established by our research group [12]. Following a 48 h period of isolation and primary culture, cellular morphology and viability were assessed, and the culture medium was replenished. After approximately 8 to 10 days (The cellular processes are prominent, the protrusions are tightly interwoven, the intricate network of nerve cells is discernible) in vitro, the cellular morphology satisfied the predetermined criteria for subsequent experimental procedures, as illustrated in Figure 3.

On the basis of the established design principles of RNAi fragments and the structural characteristics of the sheep *GRM1* gene coding sequences, we selected candidate siRNA target sequences according to the CDS regions of the *GRM1* genes available in the NCBI database. Initial screening was performed by consulting candidate fragments recommended by specialized siRNA validation and design platforms. These sequences were subsequently subjected to homology analysis against other endogenous genes using the BLAST tool (on line) on the NCBI website to preclude potential off-target effects (Table 1).

**Figure 4:**
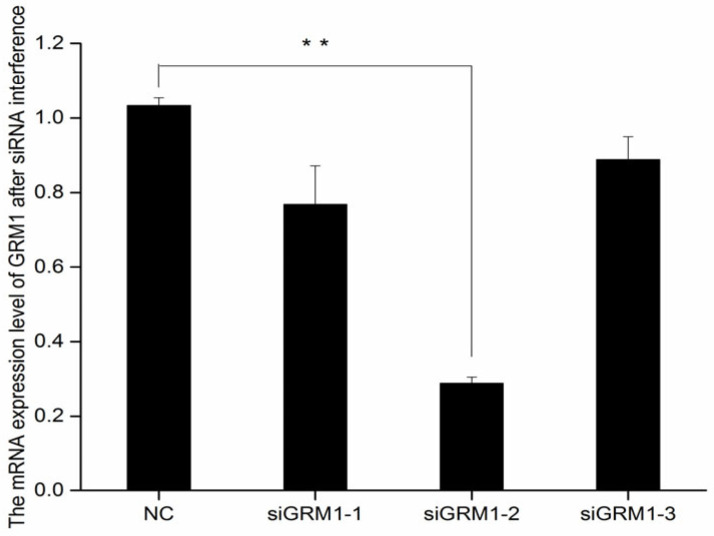
mRNA expression level after *GRM1* siRNA interference. ** *p* < 0.01.

The RNAi constructs were transfected into primary hypothalamic nerve cells isolated from fetal sheep, and the siRNA demonstrating the greatest interference efficiency was selected on the basis of quantitative evaluation. The results demonstrated that the interference efficiency of siRNA-*GRM1*-2 reached a maximum of 71% (Figure 4).

### 2.4. Transfection of Hypothalamic Nerve Cells with a GRM1 Recombinant Plasmid

**Figure 5:**
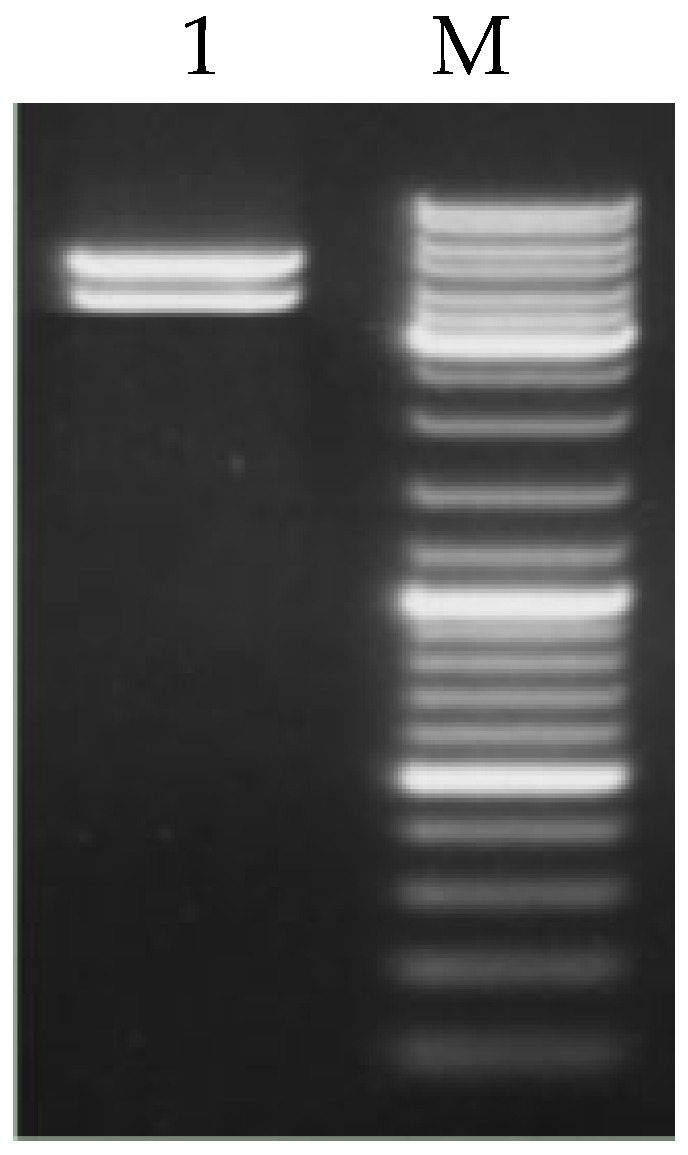
Double enzymatic digestion of the pEGFP-C2-*GRM1* vector. M is the DNA marker DL10000.

The recombinant plasmid pEGFP-C2-*GRM1* was subjected to double digestion with XhoI and BamHI restriction endonucleases to verify the successful construction of the vector (Figure 5). Electrophoretic analysis revealed that the digestion produced a DNA fragment consistent with the anticipated insert size, thereby confirming the integrity of the pEGFP-C2-*GRM1* plasmid, in accordance with the expected results.

**Figure 6:**
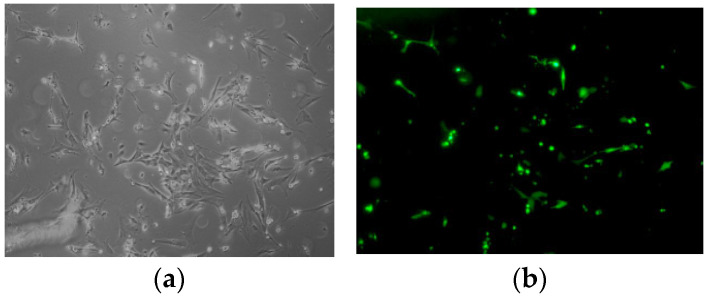
Transfection of hypothalamic nerve cells with recombinant plasmid. (**a**) Inverted microscopy image after pEGFP-C2-*GRM1* transfection. (**b**) Fluorescence image.

**Figure 7:**
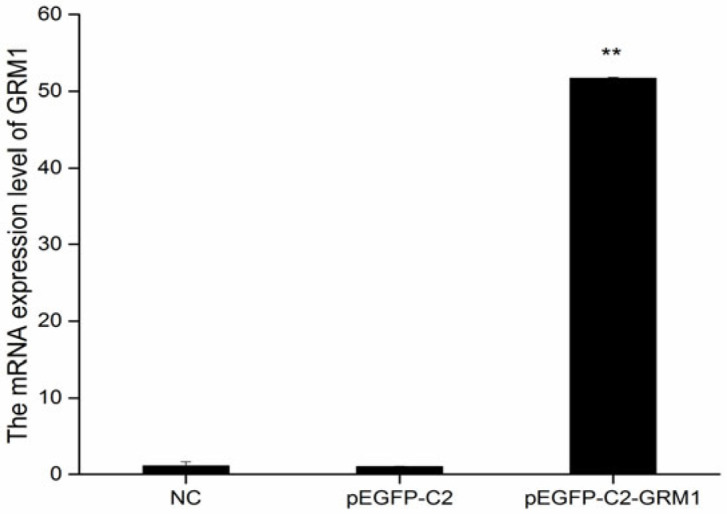
Expression of *GRM1* mRNA in hypothalamic nerve cells. ** *p* < 0.01.

The recombinant plasmid pEGFP-C2-*GRM1* was subjected to digestion with XhoI and BamHI restriction enzymes to verify the successful construction of the vector (Figure 6). Subsequently, pEGFP-C2-*GRM1* was transfected into sheep hypothalamic nerve cells via lipofection-mediated transfection, during which the optimal plasmid-to-liposome ratio was determined. The optimal transfection efficiency of pEGFP-C2, pEGFP-C2-*GRM1*, and the NC was determined at 72 h post-transfection. As shown in Figure 5, the transfected cells maintained high viability and structural integrity, accompanied by robust fluorescence signals, confirming their suitability for subsequent experimental procedures (Figure 7). Following reverse transcription of total RNA extracted from the collected cells, the expression levels of target genes and interference genes were quantified using qRT–PCR. Relative expression levels were calculated by the 2^−△△CT^ method, with β-actin serving as the internal reference gene. Statistical analysis was conducted using SPSS 17.0 (IBM Corporation, Armonk, NY, USA). All experiments were performed in triplicate for each experimental group.

### 2.5. Expression of GRM1 Protein in the Hypothalamic Nerve Cells of Sheep

**Figure 8:**
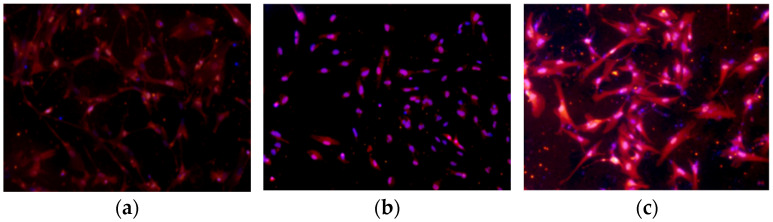
Immunofluorescence staining for the detection of *GRM1* expression after transfection. (**a**) Blank, (**b**) si*GRM1*-2, (**c**) pEGFP-C2-*GRM1*.

Immunofluorescence assays were performed to detect the expression of *GRM1* in the hypothalamic nerve cells of sheep. The results revealed that *GRM1* was widely expressed in the cells, and the expression patterns before and after interference and overexpression are illustrated in Figure 8.

### 2.6. Effects of GRM1 on Related Gene and GnRH Expression

**Figure 9:**
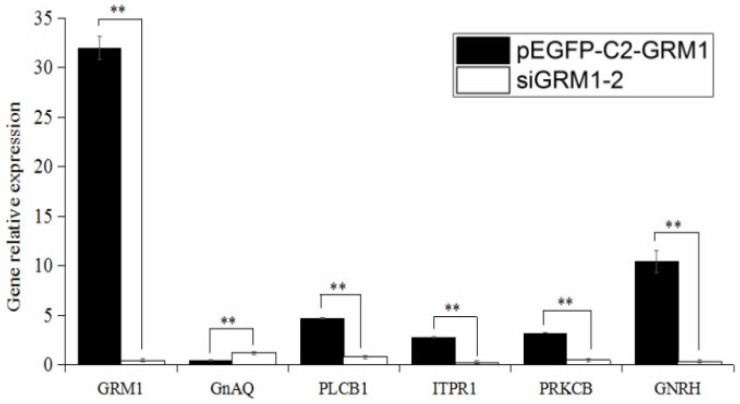
Relative expression of *GRM1* and related genes. Have already added. ** *p* < 0.01.

The expression levels of *GRM1*, GnRH, and related genes were assessed using quantitative real-time PCR following the transfection of hypothalamic nerve cells from sheep with either the recombinant plasmid pEGFP-C2-*GRM1* or siRNA-*GRM1*-2. Compared with that in the untreated group, the relative expression of *GRM1* was significantly elevated in the pEGFP-C2-*GRM1* group and markedly reduced in the siRNA-*GRM1*-2 group (*p* < 0.01; Figure 9). The expression of the downstream target gene GnAQ was significantly lower in the pEGFP-C2-*GRM1* group than in the siRNA-*GRM1*-2 group (*p* < 0.05), whereas the relative expression level of GnRH was greater in the pEGFP-C2-*GRM1* group than in the siRNA-*GRM1*-2group (*p* < 0.01). These findings suggest that *GRM1* in ovine hypothalamic nerve cells may regulate the downstream gene GnAQ through glutamatergic synapses and calcium-mediated signaling pathways, whereas GnAQ potentially modulates GnRH expression via the GnRH signaling pathway.

### 2.7. Effect of GRM1 Gene on GnRH Secretion in the Hypothalamic Nerve Cells of Sheep

**Figure 10:**
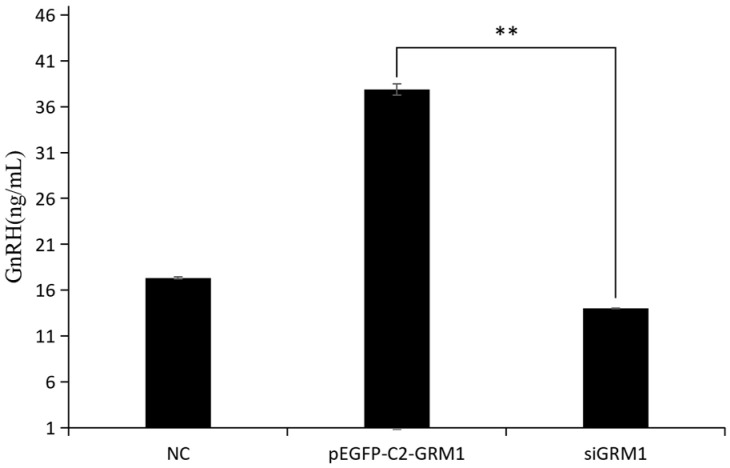
GnRH test results. ** *p* < 0.01.

The secretory levels of GnRH in ovine hypothalamic nerve cells were quantified via ELISA following transfection in both the *GRM1* overexpression and the si*GRM1*-2 group, and the results are presented in Figure 10. Compared with both the control group and the si*GRM1*-2 group, the pEGFP-C2-*GRM1* group demonstrated a marked increase in GnRH secretion (*p* < 0.05), suggesting that *GRM1* expression modulates GnRH secretion.

## 3. Discussion

The seasonal estrus in sheep is strongly governed by the HPO axis, which is synchronized with the natural photoperiod. Seasonal estrus represents an integrated physiological response through which ewes adapt to a suite of external environmental cues and photoperiodic changes. Under identical latitudinal conditions, significant variations in reproductive seasonality have been documented across various sheep breeds, as well as among individuals within the same breed. In addition to the effects of feeding management and nutritional conditions, genetic factors represent the most fundamental determinant of seasonal estrus variation. The imbalance between supply and demand in the sheep market highlights that enhancing reproductive efficiency is critical for advancing sheep production, thereby enabling ewes to produce multiple litters annually. Therefore, the ability of sheep to maintain estrus cycles throughout the year is highly important.

The consistent reproductive performance of animals is a critical factor in livestock production systems, with the regular cyclicity of estrus being essential for maintaining the operational stability of these systems. Extensive research has shown that the optimal nutritional status directly affects body weight metrics and reproductive processes in female livestock—including estrus cyclicity, mating behavior, gestation, and embryonic development—exhibiting a significant positive correlation [13,14]. Consequently, nutrition influences the growth rate and reproductive outcomes of animals, regulates the synthesis and secretion of hormones from endocrine glands, and ultimately triggers the onset of the estrus cycle [13,14,15,16,17]. Nutritional factors play a pivotal role in modulating the process of sexual maturation. An adequate nutritional status is indispensable for sustaining the physiological homeostasis of the endocrine system, given that it regulates the synthesis and secretion of hormones within the organism. During the ovulation period, ewes necessitate optimal nutritional support to sustain physiological follicular development and maturation. Ensuring an adequate provision of nutrients during this critical phase is imperative; insufficient intake may compromise follicular integrity or culminate in follicular atresia, thereby diminishing the follicular reserve. A previous study by our research group revealed that in the actual production of sheep, increasing the supply of nutrients can increase the estrus rate of Kazakh ewes to 44%, demonstrating that the level of nutrition is an important factor affecting estrus and sexual activity in sheep.

The HPO axis constitutes the central regulatory mechanism governing reproductive processes in animals, with its secreted hormones and corresponding receptor genes continuing to be a major focus of contemporary research [18]. The seasonal estrus cycle in sheep is precisely regulated by the HPO axis, which exhibits cyclic activation and suppression in response to photoperiod variations. The seasonal reproductive patterns in sheep are modulated by GnRH, which is synthesized and secreted by hypothalamic nerve cells. G protein-coupled receptors expressed on GnRH neurons constitute the principal receptor class responsible for mediating cellular signal transduction [19,20]. The differential pulse frequency of GnRH serves as a critical regulator underlying the cyclical transition between breeding and nonbreeding seasons. The pulsatile frequency of GnRH is markedly attenuated during the nonbreeding season. Consequently, if the concentration of GnRH does not exceed a critical threshold, estrus is not induced [21]. Previously, our research team performed a transcriptomic analysis of hypothalamic, pituitary, and ovarian tissues from Kazakh sheep under nutritional regulation during nonreproductive estrus, reproductive estrus, and nonreproductive anestrus, and the results revealed differential *GRM1* gene expression, implicating it in seasonal estrus regulation in sheep [12]. Ovine hypothalamic nerve cells serve as a valuable cellular model for investigating the mechanistic actions of target genes and the hormonal secretion associated with the regulation of seasonal estrus in sheep [22]. In this study, ovine hypothalamic nerve cells were used to investigate the regulatory mechanisms of the *GRM1* gene in ovine estrus and GnRH secretion.

Current research on the *GRM1* gene is predominantly centered on elucidating the pathogenic mechanisms underlying specific disorders, including psychiatric and neurodevelopmental conditions [6,23]. Glutamate (Glu) stimulates the secretion of GnRH. Following the co-culture of GnRH neurons with astrocytes, the expression of the glutamate receptor *GRM1* on GnRH neurons was significantly upregulated, and GnRH secretion was markedly enhanced [9,24,25]. Studies on the association between *GRM1* gene expression and reproductive estrus in sheep are rare. The expression of *GRM1* in the hypothalamus, pituitary gland, and ovary of Kazakh sheep during the estrus and anestrus phases was examined using qRT–PCR and immunohistochemistry. The results revealed that the expression of *GRM1* was highest in the hypothalamus and that the expression level of *GRM1* in estrus was significantly greater than that in anestrus. The immunohistochemical results revealed that *GRM1* was strongly and widely expressed in the hypothalamus; a small number of strongly positive nuclei and weak cytoplasmic expression were observed in the pituitary gland, with significantly less positive staining than in the hypothalamus; and no positive staining was detected in the ovaries. During the estrus period, *GRM1* expression was diffuse and weakly positive throughout the hypothalamus. In contrast, no nuclei displaying strong positive immunoreactivity were observed within the pituitary gland; only faint cytoplasmic immunostaining was detected, which was significantly reduced compared with that in pituitary tissue during estrus. Similarly, positive immunostaining was nearly undetectable in the ovarian tissue. Therefore, it is hypothesized that *GRM1* modulates the hypothalamic release of GnRH, consequently influencing the estrus cycle in sheep.

**Figure 11:**
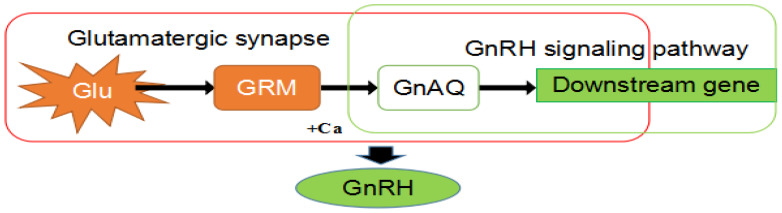
Simple pathway of *GRM1* involved in GnRH secretion.

The effects of *GRM1* expression modulation on GnRH secretion were subsequently assessed in isolated ovine hypothalamic nerve cells. This study demonstrated that the transfection of hypothalamic cells from sheep with the recombinant vectors pEGFP-C2-*GRM1* and siRNA-*GRM1*-2 resulted in the downregulation of GnAQ expression and the upregulation of GnRH secretion (Figure 10). In the glutamatergic synapse and calcium signaling pathways, the gene GnAQ, directly downstream of *GRM1*, acts on the downstream gene PLC in the GnRH signaling pathway. PLC participates in various signal transduction pathways in vivo [26]. The downstream gene PRKCB can bind to neurocalcineurin inhibitors [27], and neurocalcineurin inhibitors can reduce the activity of the HPO. It is hypothesized that PRKCB may influence cyclical changes in the ovaries by regulating the HPO, thereby altering the physiological state of the organism and inducing estrus. In the estrogen signaling pathway, E2 binds to the estrogen receptor, which subsequently dimerizes with *GRM1* [28]. This complex modulates the negative feedback mechanism of E2 on the hypothalamus, ultimately influencing the secretion of GnRH (Figure 11). Fu et al. performed comparative transcriptomic analyses on the gonadal axis of Bamei meat sheep with varying lambing rates, leading to the identification of the *GRM1* gene and the neural activity-related ligand–receptor interaction signaling pathway, it is postulated that this gene and its associated pathway may exert a pivotal regulatory influence on the modulation of the oocyte population and follicular development mediated by the gonadal axis [9]. These findings align with the experimental data obtained in this study. However, our study focuses only on Kazakh sheep, which may limit the generalizability of the findings to other breeds. Subsequent knockout and knock-in animal model experiments will be conducted to further validate mechanisms.

## 4. Materials and Methods

### 4.1. Samples

A 90-day-old female sheep fetus was obtained from a live animal slaughtering center in Shihezi, Xinjiang, select pregnant Kazakh ewes around 90 days of gestation, euthanize them to remove the uterus and fetal lambs. Choose female fetal lambs, extract the entire brain tissue under sterile conditions, and isolate the hypothalamus for neural cell culture [2]. Hypothalamic, pituitary, and ovarian tissues were collected separately from healthy adult Kazakh ewes in estrus (*n* = 3, the vulva is red with mucoid discharge, accompanied by a ram’s straddle position [29]) and non-estrus (*n* = 3), aged 2–4 years with body weights ranging from 40 to 45 ± 1.5 kg. These ewes were provided by the experimental station of Shihezi University in Xinjiang, China. This experimental procedure were strictly conducted in accordance with the Guidelines for the Care and Use of Laboratory Animals. The animal usage protocol has been reviewed and approved by the Institutional Animal Care and Use Committee of the First Affiliated Hospital of Shihezi University School of Medicine, Xinjiang, China (Approval No.: A2020-107-01).

### 4.2. Primer Design for RT–qPCR and mRNA Expression Analysis

RNA was extracted from hypothalamic, pituitary, and ovarian tissues using the TRizol method. and reverse transcription was performed to obtain cDNA with the Prime-Script™ RT Kit (including gDNA Eraser, Taraka, Dalian, China). Primer design was performed using Primer5.0, with the primers meeting the requirements for real-time fluorescence quantitative PCR, and the NCBI online tool was used to determine the specificity of the primers. The primer information for sheep *GRM1* and β-actin is provided in Table 2 (provided by Shanghai BioSynthetic, Shnghai, China). The expression levels of *GRM1* gene mRNA in hypothalamic, pituitary, and ovarian tissues were detected using Light Cycler 96 Real-Time Quantitative PCR System by the FastStart Universal SYBR Green Master (Rox), with three replicates per group. The β-actin as the internal reference gene. Real-time quantitative PCR reaction system: SYBR Green Master 10 μL, Upstream and downstream primer 1 μL, cDNA 1.5 μL, ddH_2_O 6.5 μL, Total 20 μL. reaction condition: 95 °C for 600 s, 95 °C for 30 s, 57 °C for 30 s, and 72 °C for 30 s for 55 cycles.

### 4.3. Immunohistochemical Detection of GRM1 in the Hypothalamus, Pituitary Gland, and Ovary

Preparation of paraffin-embedded tissue sections: Fixation: Hypothalamic, pituitary, and ovarian tissues were immersed in 4% paraformaldehyde for a minimum of 48 h. Dehydration: Following fixation, the tissues were rinsed under running water overnight and then sequentially dehydrated in 70%, 80%, and 90% ethanol for 30 min each, followed by two 20 min treatments in 95% and absolute ethanol. Clearing: Tissues were incubated in a 1:1 mixture of absolute ethanol and xylene for 15 min, followed by two separate 15 min incubations in xylene I and xylene II. Embedding: Samples were transferred to a xylene–paraffin mixture for 50–60 min and then immersed in pure paraffin I and paraffin II, each for 50–60 min, before final embedding in paraffin blocks. The wax block was continuously sectioned at approximately 4–6 μm, spread out, mounted, labeled, and then baked in a 62 °C oven for 2 h for subsequent use.

*GRM1* immunohistochemical staining procedure: After the sections were heated at 65 °C for 30 min, they were dewaxed with xylene and an alcohol gradient to water, fixed in boiling citric acid solution for 8 min, and then cooled to room temperature. The sections were then immersed in 3% H_2_O_2_ at room temperature in the dark for 10 min, followed by three rinses with water. The sections were rinsed with PBS three times for 5 min each and blocked with goat serum at room temperature for 30 min. Primary antibody (CST, rabbit monoclonal *GRM1* antibody, 12,551) was added at a dilution of 1:1000 (the optimal concentration determined by titration), and the samples were incubated in a wet box at 4 °C overnight. The next day, the samples were extracted and incubated at 37 °C for 30 min, after which the secondary antibody (zsbio, goat anti-rabbit IgG, Polymers, ZB-2306) was added, and the samples were incubated at 37 °C for 30 min. Under DAB microscopy, the color was observed. Then, the sections were restained with Sumatine for 1–2 min, rinsed with tap water, fixed with acetic alcohol for several seconds, and rinsed with tap water again. Subsequently, gradient alcohol dehydration, clearing, drying, neutral gum sealing, and microscopic observation were performed. Note: After the primary and secondary antibodies were added and each staining step was completed, the sections were rinsed with PBS three times for 5 min each.

### 4.4. Design and Screening of siRNA Fragments for the GRM1

On the basis of the coding DNA sequence (CDS) region of the *GRM1* gene published by NCBI, the design principles for RNAi fragments, and the characteristics of the sheep *GRM1* gene coding sequence, we initially screened and obtained the siRNA target sequence by referencing the fragments provided by professional siRNA interference effect validation and screening design websites. BLAST analysis was performed on the NCBI website to assess the homology of these sequences with those of other genes in vivo, ensuring that no interference with other genes occurred.

### 4.5. Construction of the pEGFP-C2-GRM1 Overexpression Vector

The complete CDS of the sheep *GRM1* gene (containing XhoI and BamHI restriction enzyme sites) was provided to Shanghai Synthetic Biology (Shanghai, China). The target fragment product was ligated to the pEGFP-C2 plasmid to construct the pEGFP-C2-*GRM1* expression vector. The plasmids were extracted and digested with the restriction enzymes XhoI and BamHI for identification. The recombinant plasmid pEGFP-C2-*GRM1* was used for large-scale endotoxin extraction (refer to the instructions in the Tiangen Plasmid Endotoxin Removal Kit, DP120, Beijing, China), followed by concentration determination, aliquoting, and storage at −20 °C for subsequent cell transfection.

### 4.6. Transfection of siRNA-GRM1 and pEGFP-C2-GRM1 into the Hypothalamic Nerve Cells of Sheep

The isolation of primary hypothalamic nerve cells from sheep was performed according to a previously reported method [10]. This study was approved by the Experimental Animal Ethics Committee of Shihezi University (approval number A2020-107-01). Briefly, sheep hypothalamic tissue was carefully decorticated, minced, and digested with collagenase IV. After the digestion was terminated, the tissue was centrifuged, and the cell pellet was resuspended in neurobasal medium-A (Gibco) supplemented with 2% B27 (Gibco). The suspension was gently pipetted and mixed and then seeded onto polylysine-coated culture dishes. After 48 h, the medium was replaced with neurobasal medium-A, B27, and 100×GlutaMAX (100:2:1). Neuronal identification was performed using Cellular Immunofluorescence Method.

Transfection of hypothalamic nerve cells in sheep: pEGFP-C2-*GRM1* transfection groups: NC, pEGFP-C2, and pEGFP-C2-*GRM1*. After 24 h, the expression of green fluorescent protein (GFP) in the cells was observed using an inverted fluorescence microscope (TE2000; Nikon, Tokyo, Japan) to evaluate the transfection efficiency. Three replicates per group were used.

### 4.7. qRT–PCR-Based Detection of the Impact of GRM1 on Genes Within the Estrus-Related Signaling Pathway

Cells were harvested 48 h post-transfection. Total RNA was extracted from each experimental group using the TRIzol method, and RNA quality and concentration were determined by spectrophotometry. cDNA synthesis was subsequently performed according to the manufacturer’s instructions in the Taraka Reverse Transcription Kit (RR014, Dalian, China).

cDNA from the NC, pEGFP-C2-*GRM1*, and siRNA-*GRM1*-2 groups was used. qRT–PCR was used to measure the mRNA expression levels of *GRM1*, GnAQ (G protein subunit αq), ITPR1 (inositide 1,4,5-trisphosphate receptor type 1), PLCB1 (phospholipase Cβ1), and PRKCB (protein kinase Cβ) in each group (Table 3). The internal reference gene was β-actin. Each reaction was performed in triplicate, and data analysis was conducted using the 2^−ΔΔCT^ method.

### 4.8. Immunofluorescence-Based Detection of GRM1 Protein Expression After Transfection

The expression of *GRM1* protein in the NC, si*GRM1*, and pEGFP-C2-*GRM1* groups was detected after transfection. Identification was performed by immunofluorescence staining with an anti-GRM1 antibody (1:100).

### 4.9. Detection of GnRH Secretion in Hypothalamic Nerve Cells

The culture medium of hypothalamic nerve cells transfected with pEGFP-C2-*GRM1* and si*GRM1*-2 for 48 h was collected, as was the culture medium of nontransfected cells. The GnRH concentration in the culture medium was determined using a sheep GnRH ELISA kit (BLUEGENE, Shanghai, China). The procedures were performed strictly according to the kit instructions. The optical density (OD) at 450 nm was detected using a microplate reader, and the GnRH concentration in each sample group was calculated using a standard curve.

### 4.10. Statistical Analysis

RNAi or overexpression of *GRM1* was used to determine relative changes in expression compared with that in the control group, and the negative control (NC) was used as the control group. The 2^−∆∆CT^ method was used to analyze expression levels via qRT–PCR. Data are presented as the mean ± SEM of at least three independent replicates. *p* < 0.05 indicates a statistically significant difference; *p* < 0.01 indicates an extremely significant difference.

## 5. Conclusions

Seasonal estrus limits sheep farming efficiency, making enhanced reproductive capacity key for breeding. *GRM1*, a neuronal modulator, was identified as a candidate gene regulating estrus in Kazakh sheep. Expression levels of *GRM1* mRNA and protein were measured via PCR and immunohistochemistry during anestrus and nutritionally induced estrus, showing increased *GRM1* in estrus. Cellular experiments using overexpression and RNA interference revealed that *GRM1* upregulation affects genes like GnAQ, ITPR1, PLCB1, and PRKCB, promoting GnRH secretion, while *GRM1* interference reduced GnRH. *GRM1* modulates GnRH secretion in hypothalamic neurons through the glutamatergic synapse–calcium signaling pathway.

## Figures and Tables

**Table 1:** *GRM1* interference fragments.

siRNA	siRNA Sequence (5′–3′)
siRNA-*GRM1*-1	GCAUUCUCACAGCCUGUUUTT
	AUAGGUCCAAUUGUACCGCTT
siRNA-*GRM1*-2	GCAGCCAAACUCCUGGAAUUTT
	AAUUCCAGGAGUUUGCUGCTT
siRNA-*GRM1*-3	CCAAACAGCCGUCAUCAAATT
	UUUGAUGACGGCUGUUUGGTT

**Table 2:** Primer sequences for quantitative real-time PCR.

Gene Name	Primer Sequence (5′–3′)	Tm/°C	Length (bp)
*GRM1* (ENSOARG00020084779)	CCTACCCGAGCATCAAAGAAG	57	217
	TCCAAAGTAAATGGGCACGAAA		
*β-Actin* (ENSOARG00020008714)	AGAGCAAGAGAGGCATCC	50–68	108
	TCGTTGTAGAAGGTGTGGT		

**Table 3:** Primer information for qRT–PCR.

Gene Name	Primer Sequence (5′–3′)	Tm/°C	Length (bp)
*GRM1* (ENSOARG00020084779)	CCTACCCGAGCATCAAAGAAGT	57	217
	CCAAAGTAAATGGGCACGAAA		
*GnAQ* (ENSOARG00020004509)	GAGAACCGAATGGAGGAAAG	54	144
	GAAATAGTCAACTAGGTGGGAAT		
*ITPR1* (ENSOARG00020012519)	GTCCGCCACCAGTTCAA	54	134
	CCTCTGCTGCTAAATAATGC		
*PLCB1* (ENSOARG00020023836)	TTTTCCGAATTTGGTGC	54	171
	GGCGAGGCTGTTGTTAG		
*PRKCB* (ENSOARG00020025105)	GATCGAACTACACGGAACG	54	203
	TAGAGGCTCAGTGGTAAAGAAT		
*β-Actin* (ENSOARG00020008714)	AGAGCAAGAGAGGCATCC	50–68	108
	TCGTTGTAGAAGGTGTGGT		

## Data Availability

The original contributions presented in this study are included in the article. Further inquiries can be directed to the corresponding author.

## Abbreviations

The following abbreviations are used in this manuscript:

*GRM1*	metabotropic glutamate receptor 1
*GnRH*	Gonadotropin-releasing hormones
*RNAi*	RNA interference
*HP**O*	Hypothalamic–Pituitary–Ovarian
*CDS*	Coding DNA Sequence
*GnAQ*	G Protein Subunit αq
*ITPR1*	Inositide 1,4,5-trisphosphate Receptor Type 1
*PLCB1*	Phospholipase Cβ1
*PRKCB*	Protein Kinase Cβ
